# Key CT and MRI findings of drug-associated hepatobiliary and pancreatic disorders

**DOI:** 10.1007/s11604-023-01505-z

**Published:** 2023-11-06

**Authors:** Shintaro Ichikawa, Satoshi Goshima

**Affiliations:** Department of Radiology, Hamamatsu University School of Medicine, Hamamatsu, Shizuoka Japan

**Keywords:** Drug-induced abnormalities, Multidetector computed tomography, Magnetic resonance imaging, Sinusoidal obstruction syndrome, Chemotherapy

## Abstract

Obtaining an imaging diagnosis of various hepatobiliary and pancreatic disorders caused by certain drugs can often be challenging. Familiarity with these conditions may improve diagnostic accuracy and patient management. This review aimed to describe the imaging findings of drug-associated hepatobiliary and pancreatic disorders and identify suggestions for obtaining a correct diagnosis. We focused on relatively common disorders or those that can present with characteristic imaging findings, such as drug-induced acute hepatitis, sinusoidal obstruction syndrome, focal nodular hyperplasia-like lesions, hepatocellular adenoma, pseudocirrhosis, chemotherapy-associated steatohepatitis, amiodarone deposition in the liver, secondary iron overload, drug-induced pancreatitis, pancreatic enlargement after epoprostenol therapy, ceftriaxone-associated gallbladder pseudolithiasis, immune-related adverse events, and methotrexate-associated lymphoproliferative disorders.

## Introduction

Since most drugs are metabolized in the liver, liver injury is an inevitable consequence of drug exposure. The temporal relationship between the drug administration and the appearance and resolution of liver injury, as well as the exclusion of other causes, is crucial in the diagnosis of drug-induced liver injury (DILI). Most DILI occurs within 60 days of taking drugs, but onset after 90 days is also observed. Allergic cases may develop within 24 h [1]. The first-choice imaging modality for a patient with suspected DILI is abdominal ultrasonography. The choice of additional abdominal imaging is significantly influenced by the patient’s symptomatology. If abdominal pain is a prominent symptom, additional imaging tests, such as computed tomography (CT) and magnetic resonance imaging (MRI), may be necessary despite a normal abdominal ultrasound to exclude gallstone disease and other competing etiologies, including tumors [1]. CT and MRI are valuable tools to accurately and reproducibly diagnose hepatobiliary and pancreatic abnormalities. The choice between the recommended imaging protocols depends on the patient’s age and diseases and cannot be prescribed in general. For example, to rule out cholangitis and tumors, non-contrast and contrast-enhanced CT are recommended, and dynamic CT may be acceptable for the initial examination. If cholangiopathy is suspected, magnetic resonance cholangiopancreatography (MRCP) should be added to the routine MRI sequence. Alternatively, for assessing fatty or iron deposits in the liver, non-contrast CT or MRI may suffice. Pharmacotherapy is still evolving, and new agents are being introduced into routine practice, including molecular-targeted drugs and immune checkpoint inhibitors (ICIs). Drugs are essential for treating various diseases, but physicians can encounter various drug-related abnormalities. In particular, drug-associated disorders can occur in the pancreaticobiliary system, albeit less frequently than in the liver. Imaging findings of drug-associated hepatobiliary and pancreatic disorders are often normal, making diagnoses challenging in rare cases. However, certain disorders exhibit characteristic imaging findings or patient histories. Therefore, familiarity with the characteristic findings in these cases can enhance diagnostic precision and patient care. This article aimed to describe the CT and the MRI findings of drug-associated hepatobiliary and pancreatic disorders and identify tips for determining a correct diagnosis.

### Liver

#### Drug-induced acute hepatitis

Although rare, it is essential to identify significant causes of liver disease. Various pharmaceuticals, natural remedies, and dietary supplements can induce liver injury. Liver diseases can range from modest elevations in serum liver enzyme levels to fatal outcomes. Using the earliest detected increase in liver enzyme levels, three DILI patterns are identified: hepatocellular pattern: if alanine aminotransferase (ALT) is elevated five-fold above the upper limit of normal (ULN) or ALT/alkaline phosphatase (ALP) ratio (*R*) ≥ 5; cholestatic pattern: ALP is elevated two-fold above ULN or *R* ≤ 2; and mixed pattern: *R* > 2 to < 5 [1].

**Fig. 1:**
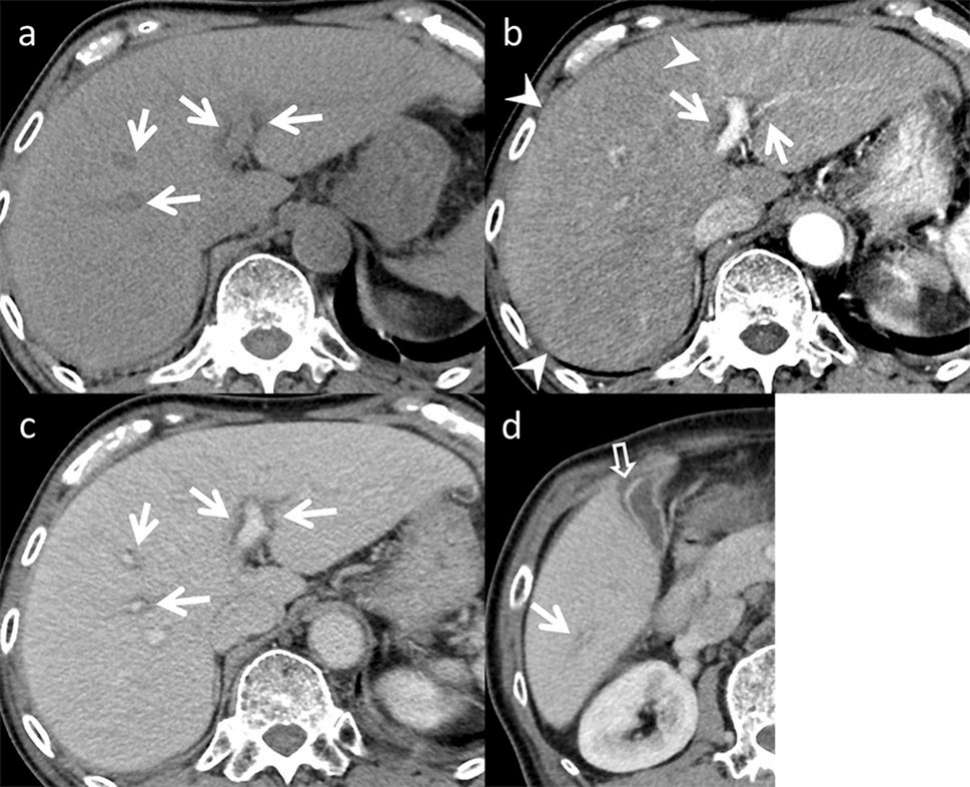
Drug-induced acute hepatitis in an 81-year-old man with appetite loss and hepatobiliary enzyme elevation, who was taking tizanidine for 1 month. Dynamic computed tomography (CT) (**a** pre-contrast, **b** arterial phase, and **c** portal venous phases) demonstrates periportal edema (arrows). **b** The liver parenchyma is heterogeneously enhanced in the arterial phase (arrowheads). **d** The gallbladder wall is thickened in the portal venous phase (open arrow). However, the gallbladder is not enlarged or distended, differentiating it from acute cholecystitis. The hepatobiliary enzyme levels normalized after tizanidine withdrawal. On imaging, acute hepatitis from other causes is differential; however, both are nonspecific and difficult to distinguish on imaging alone

Acetaminophen (paracetamol) consumption is the most prevalent cause of drug-induced hepatitis [2]. An elevated international normalized ratio of prothrombin time is a more reliable indicator of liver dysfunction than aspartate aminotransferase and ALT levels [3]. Since there are no specific imaging findings for drug-induced acute hepatitis, it is essential to exclude more common causes when diagnosing this condition, including viral infections, alcohol abuse, and autoimmune conditions. When treating patients with liver injury, the primary approach involves immediate discontinuation of the offending drug [3]. An example of this is the case of a patient presenting with hepatosplenomegaly. Reduced echogenicity and CT attenuation are observed in the liver parenchyma. The liver parenchyma exhibits heterogeneous enhancement during arterial phase imaging (Fig. 1). The observed periportal edema displays hypoattenuation on CT and hyper-intensity on T2-weighted images. Furthermore, gallbladder wall thickening is present, specifically subserosal edema, without distention. Ascites and lymph node enlargement are detected in the hilar region [4]. These findings are observed in the hepatocellular pattern DILI. Cholestatic pattern DILI may cause sclerosing cholangitis-like changes [1], and MRCP provides a comprehensive image of the biliary tree. In chronic cases, varying degrees of intrahepatic bile duct loss may occur, known as vanishing bile duct syndrome [5]. The mixed type can present with imaging findings similar to those observed in the hepatocellular and cholestatic pattern, depending on severity and course of the disease.

#### Sinusoidal obstruction syndrome (SOS)

**Fig. 2:**
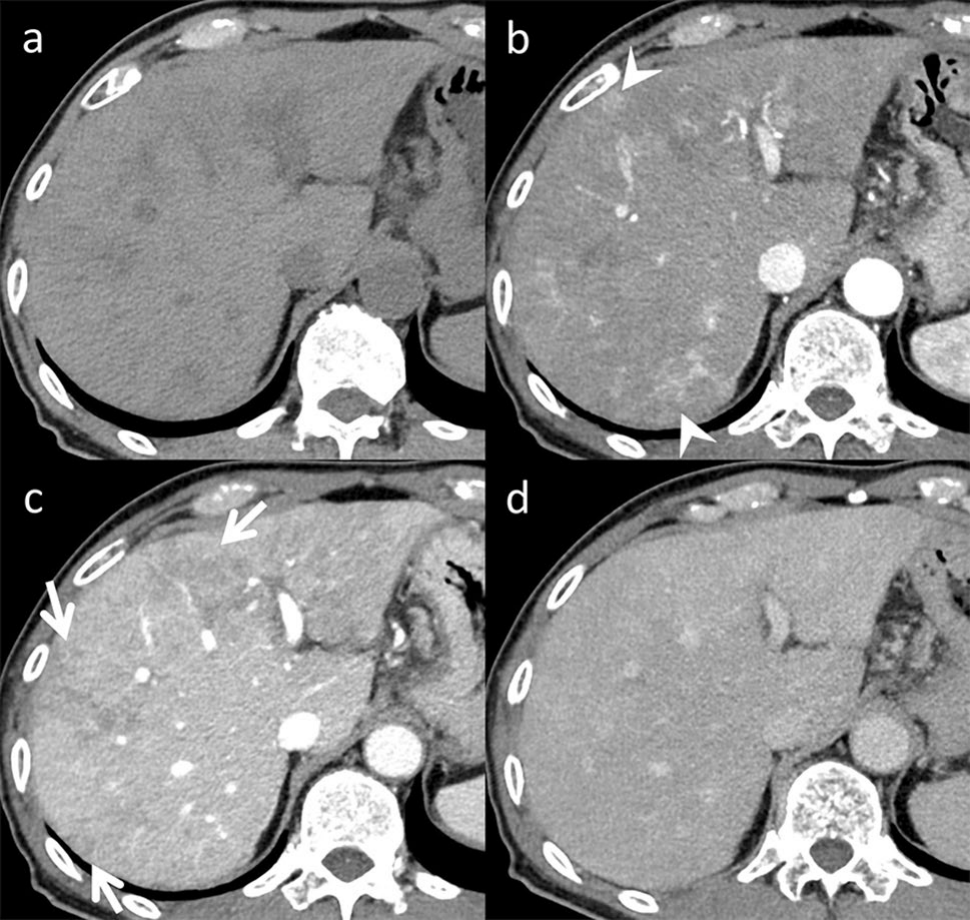
Sinusoidal obstruction syndrome in an asymptomatic 65-year-old man, who was undergoing chemotherapy (S-1 and oxaliplatin) for esophagogastric junction cancer. Dynamic computed tomography (CT) (**a** pre-contrast, **b** arterial phase, **c** portal venous phase, and **d** equilibrium phase) demonstrates diffuse reticular hypoattenuation in the liver, most obvious in the portal venous phase. These areas are unclear on pre-contrast and equilibrium phase images. Patchy heterogeneous enhancement is also observed in the arterial phase

**Fig. 3:**
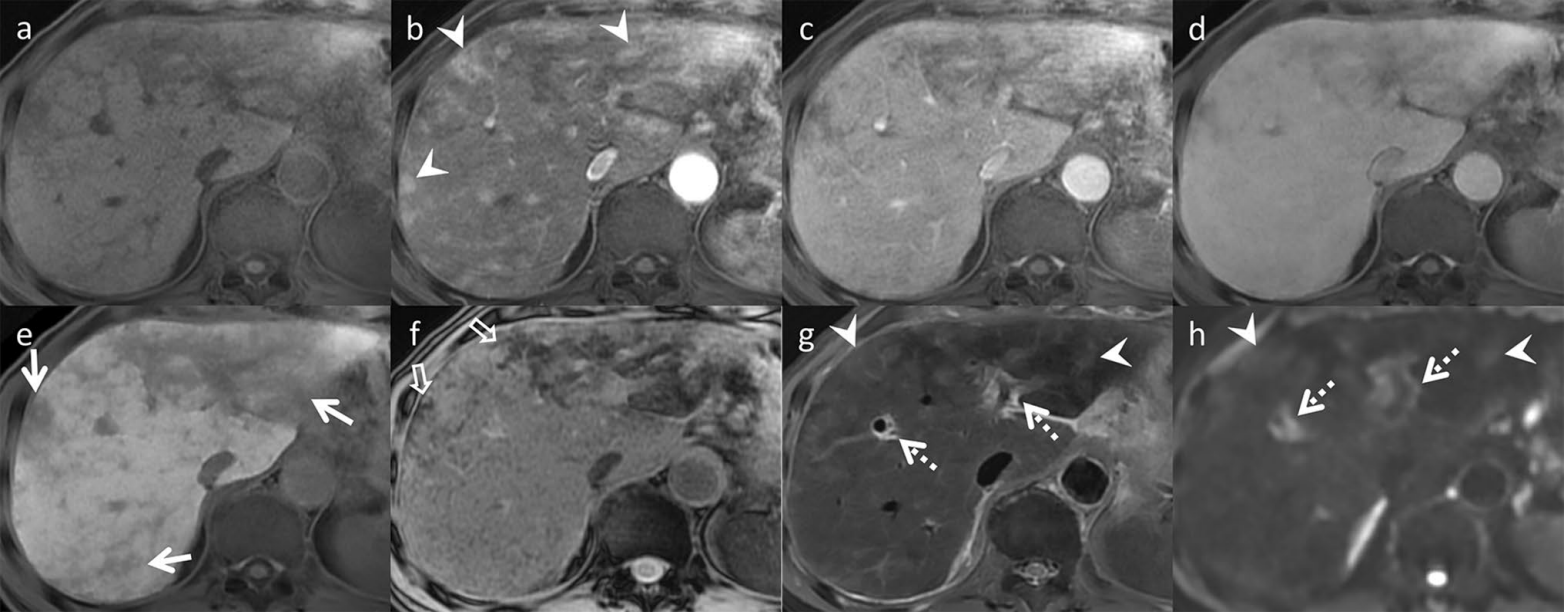
Sinusoidal obstruction syndrome (SOS) in an asymptomatic 65-year-old man, who was undergoing chemotherapy (S-1 and oxaliplatin) for esophagogastric junction cancer (same patient in Fig. 2). Gadoxetic acid-enhanced magnetic resonance imaging (MRI) (**a** pre-contrast, **b** arterial phase, **c** portal venous phase, **d** transitional phase, and **e** hepato-biliary phase) demonstrates diffuse hypo-intense reticulations in the liver, most obvious in the hepatobiliary phase (arrows). f. These areas show hypo-intensity on T2*-weighted imaging, which indicates hemorrhage (open arrows). Patchy heterogeneous enhancement in the arterial phase, heterogeneous hyper-intensity on g. fat-saturated T2-weighted imaging (fat-sat. T2WI), and h. diffusion-weighted imaging (DWI, arrowheads) with b value 800 s/mm^2^ are also evident. Periportal hyper-intensity is also observed in fat-sat. T2WI and DWI (dotted arrows). MRI is superior to computed tomography in the detection of SOS

**Fig. 4:**
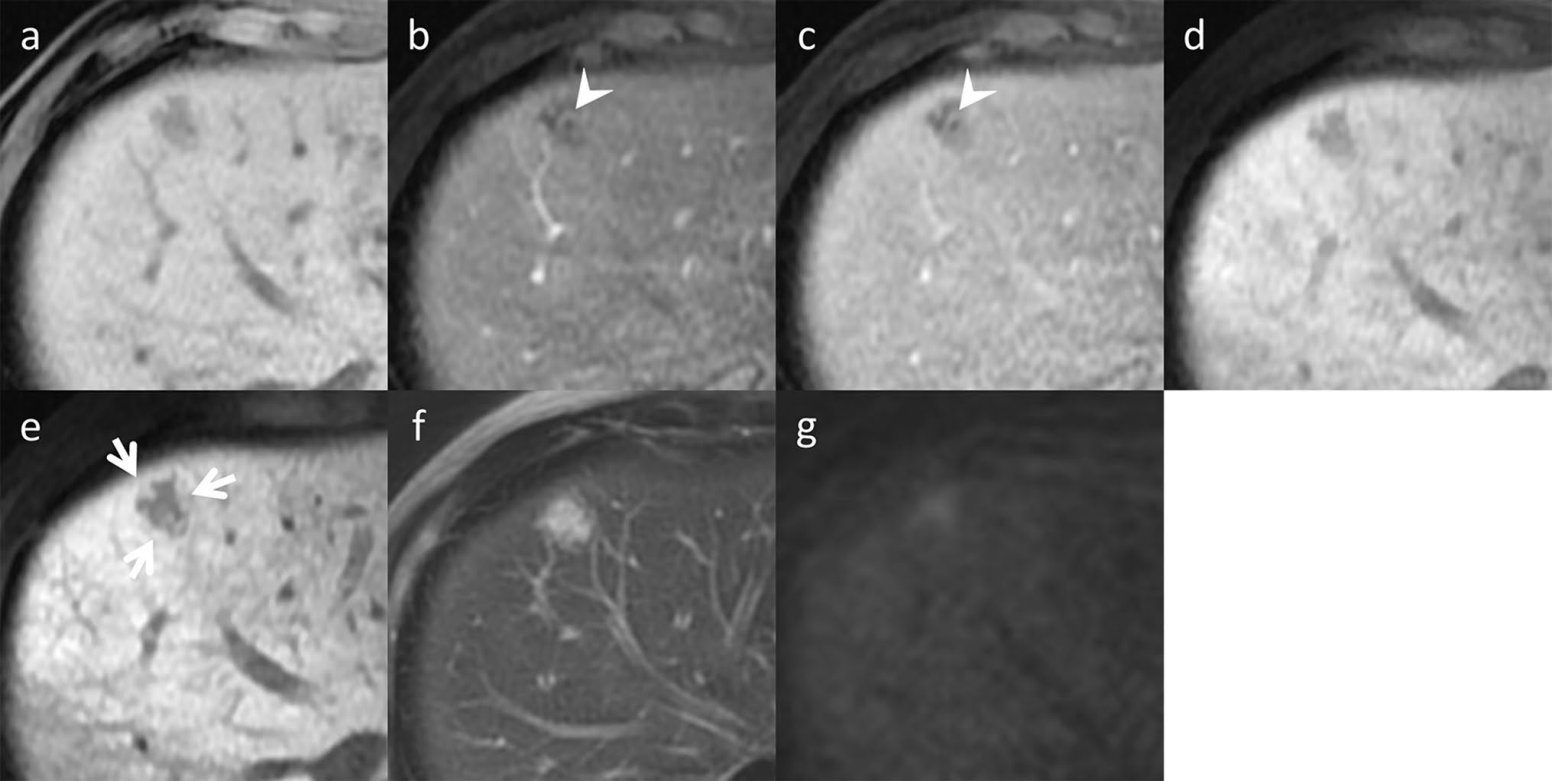
Sinusoidal obstruction syndrome (SOS) in an asymptomatic 49-year-old man, who was undergoing chemotherapy, including oxaliplatin for colon cancer. Gadoxetic acid-enhanced magnetic resonance imaging (MRI) (**a** pre-contrast, **b** arterial phase, **c** portal venous phase, **d** transitional phase, and **e** hepatobiliary phase) demonstrates a hypo-vascular lesion at S4/8, which demonstrates hyper-intensity on f. T2-weighted imaging and g. diffusion-weighted imaging with b value 800 s/mm^2^. The apparent diffusion coefficient value of this lesion is not reduced (data not shown). Peripheral gadoxetic acid uptake is observed in the hepatobiliary phase (arrows) and intra-lesional vessels in the arterial phase as well as the portal venous phase (vessel-penetrating sign, arrowheads). These are key imaging findings that differentiate mass-forming SOS from liver metastases

**Fig. 5:**
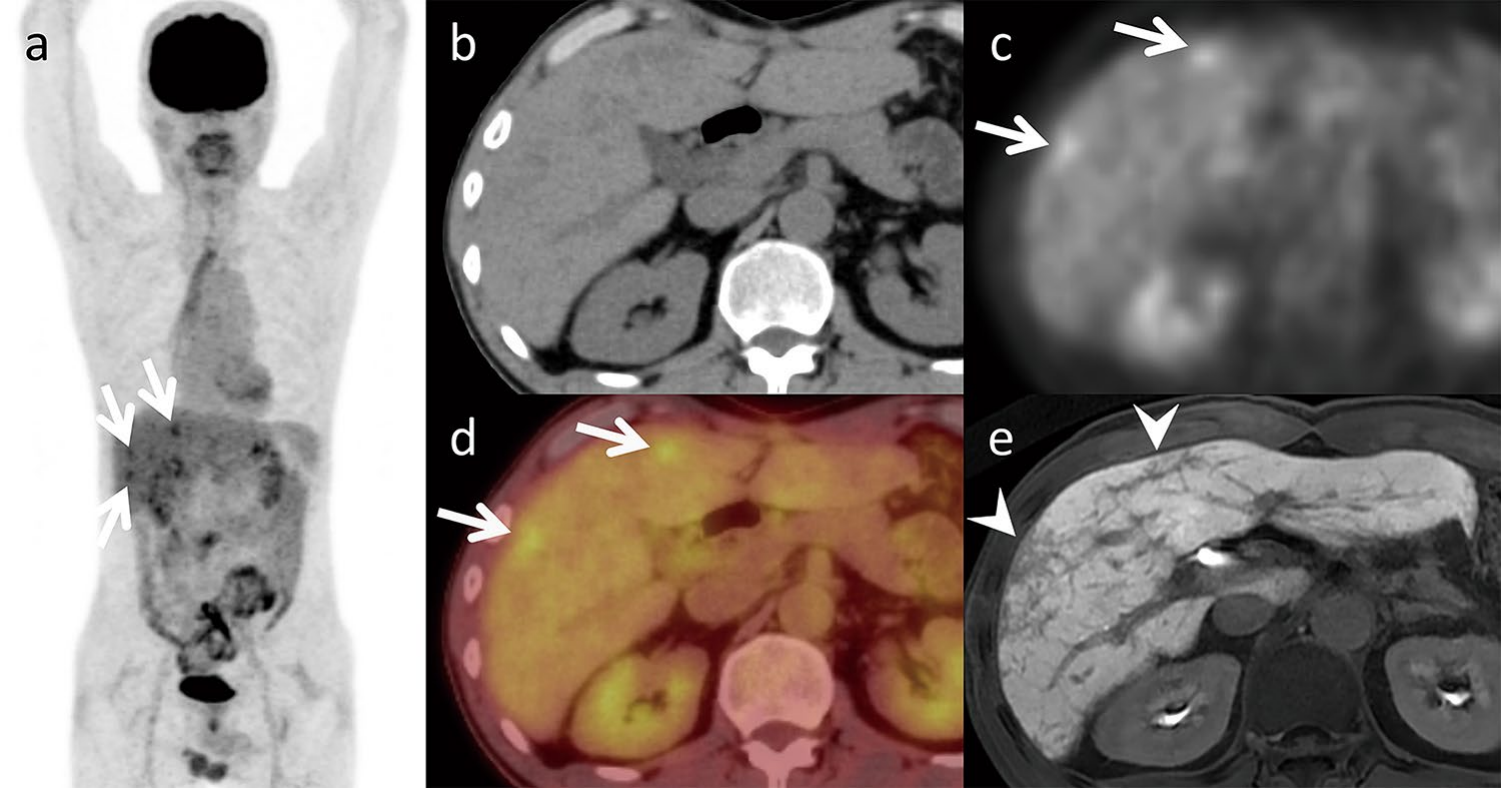
Sinusoidal obstruction syndrome (SOS) in an asymptomatic 52-year-old man, who was undergoing chemotherapy (S-1) for esophageal cancer. Fluorodeoxyglucose positron emission tomography (FDG-PET) (**a** maximum intensity projection of FDG-PET, **b** computed tomography (CT), **c** FDG-PET image, and **d** fused FDG-PET/CT image) demonstrates several focal tracer uptakes (maximum standardized uptake value, 4.0–5.0) in the liver (arrows). Multiple liver metastases were suspected; however, the hepatobiliary phase demonstrates reticular hypo-intensity in the liver (arrowheads), typical of SOS

SOS was previously known as veno-occlusive disease and is often called “blue liver.” The primary etiology of this condition is damage to the hepatic sinusoidal endothelium due to toxic exposure [6]. Oxaliplatin is regarded as the most significant SOS contributor and is primarily associated with its development. Other chemotherapeutic agents can also induce SOS, including S-1 and cisplatin [7]. Symptoms of SOS are typically nonspecific, which makes diagnosis difficult. Several risk factors increase the likelihood of developing SOS, including preoperative gamma-glutamyl transferase elevation, advanced age, female sex, increased indocyanine green retention, the number of chemotherapy cycles, and a brief interval between the end of chemotherapy and digestive surgery [6]. SOS increases the risk of perioperative as well as postoperative bleeding and hepatic failure. The primary treatment strategy for halting SOS is the immediate cessation of chemotherapy [6]. SOS imaging findings are liver enhancement with a mosaic appearance, typically observed in the peripheral parenchyma of the right lobe during the arterial phase (Fig. 2). Gadoxetic acid-enhanced MRI is a sensitive and highly specific diagnostic tool with 75% sensitivity and 96–100% specificity [4, 8]. During the hepatobiliary phase, hypo-intense reticular hepatic parenchyma is observed (Fig. 3). This condition may manifest as a new focal lesion that mimics liver metastasis [4]. SOS is differentiated from metastases by considering the absence of restricted diffusion, peripheral gadoxetic acid uptake, and intra-lesional vessels (Fig. 4). Damage to sinusoidal endothelial cells in SOS is particularly severe in zone 3 around the central vein, where the expression of the organic anion transporting polypeptide 1B3 (OATP1B3), an uptake transporter of gadoxetic acid, is high. Since the OATP1B3 expression in this region is significantly reduced in SOS, the uptake of gadoxetic acid is impaired in SOS because the OATP1B3 expression in this region is significantly decreased [9]. This may be related to peripheral gadoxetic acid uptake of focal SOS. The ability of SOS to absorb ^18^F-fluorodeoxyglucose (FDG) is a potential pitfall, which may contribute to confusion or misinterpretation [10] (Fig. 5).The mechanism for FDG uptake of SOS remains unclear. Kim et al. speculated that in patients with SOS, microcirculatory disturbances caused by endothelial cell injury and blood stasis in peliosis may lead to hepatic congestion, resulting in passive increase in blood-pool FDG tracer activity in the liver [10]. A similar mechanism may contribute to the accumulation of FDG in SOS lesions.

#### Mass formation

##### Focal nodular hyperplasia (FNH)-like lesions

**Fig. 6:**
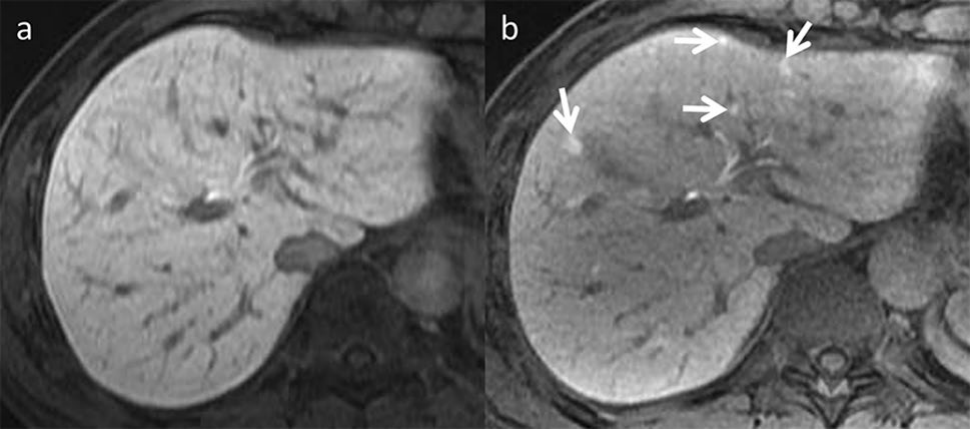
Focal nodular hyperplasia** (**FNH)-like lesions in an asymptomatic 63-year-old man, who was undergoing chemotherapy (CTX) for descending colon cancer, including oxaliplatin. **a** Pre-CTX hepatobiliary phase imaging reveals no focal liver lesions. **b** Small hyperintense lesions are observed in bilateral liver lobes during the hepatobiliary phase imaging performed six months post-initial CTX (arrows). Although they are newly arising nodules during follow-up, they can be differentiated from liver metastases by their high signal in the hepatobiliary phase imaging

FNH-like lesions are considered end-stage post-chemotherapy SOS [11]. Liver injury induces hyperplasia of periportal hepatocytes. Multiple lesions are typically observed. After completing chemotherapy, the average time between diagnosis and treatment is 48 months. The number of lesions detected by imaging may increase by up to 42 percent [4]. The lesions exhibit homogenous arterial phase hyperenhancement and iso-attenuation or iso-intensity during the portal venous as well as equilibrium phases. The lesions appear hyperintense during the hepatobiliary phase (Fig. 6) in their entirety or a doughnut-like pattern [4, 11, 12]. Although no coherent case series has been reported in adults, a study of 16 cases in pediatric cancer patients reported a median maximum diameter of 19.5 mm (range 8–41 mm) for FNH-like lesions that developed after chemotherapy [13].

##### Hepatocellular adenoma (HCA)

**Fig. 7:**
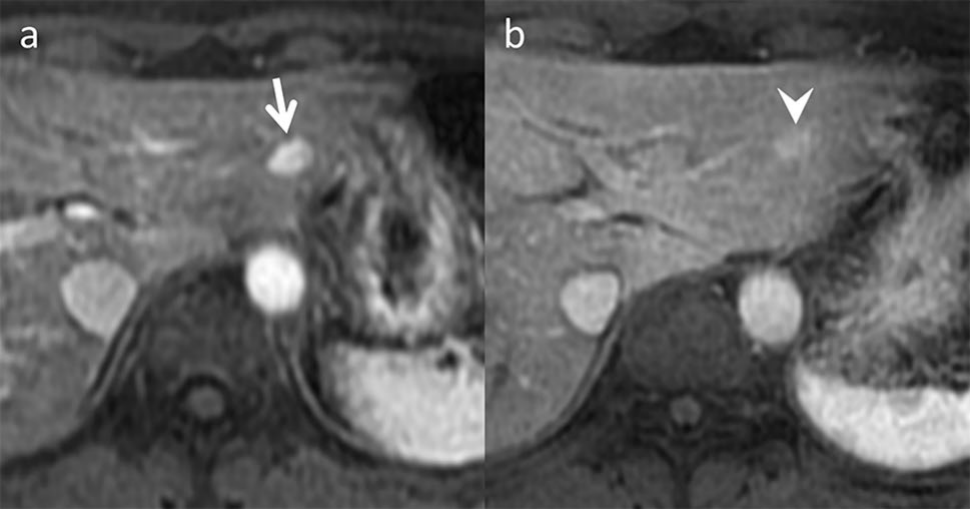
Hepatocellular adenoma in an asymptomatic 30-year-old woman, who was taking oral contraceptives. **a** Arterial phase of gadoxetic acid-enhanced magnetic resonance imaging (MRI) reveals a hyper-vascular lesion (9 mm in diameter) in segment 2 in the liver (arrow). **b** This lesion had shrunk (6 mm in diameter) in the arterial phase of gadoxetic acid-enhanced MRI after oral contraceptives were discontinued (arrowhead). Hepatocellular carcinoma is raised as a differential on imaging; however, it can be differentiated by age and the absence of chronic liver disease

HCA is a well-known drug-related hepatic mass frequently associated with women of reproductive age who take oral contraceptives containing exogenous estrogens. HCA can also occur in patients treated with anabolic steroids for athletic enhancement, Fanconi anemia, and aplastic anemia [14]. The annual incidence of HCA among oral contraceptive pill users ranges between 30 and 40 cases per million, compared to 1 to 1.3 cases per million among nonusers. This risk increases with continued use beyond 5 years [14]. HCAs are classified into four main subtypes: hepatocyte nuclear factor 1α inactivated HCA (H-HCA), inflammatory HCA (I-HCA), β-catenin activated HCA, and unclassified HCA. H-HCA and I-HCA are more common in women taking oral contraceptives [15]. A study of 78 HCA patients revealed that discontinuing oral contraceptives caused 37.2% of HCA to regress by 30% and 5.1% to regress completely [16]. Marked fat deposition, resulting from impaired fat metabolism, is the histologic hallmark of H-HCA. The most prominent imaging feature is the decreased signal in the opposed-phase compared to the in-phase chemical shift imaging (CSI). Dynamic studies revealed mild to moderate enhancement in the arterial phase and washout in the portal venous phase. H-HCA exhibits hypo-intensity on hepato-biliary phase imaging [17, 18] (Fig. 7).

#### Diffuse parenchymal change

##### Pseudocirrhosis

**Fig. 8:**
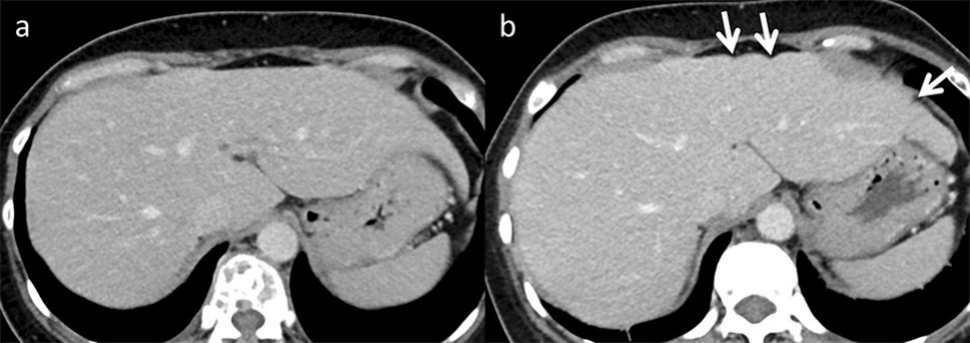
Pseudocirrhosis in an asymptomatic 39-year-old woman, who was undergoing chemotherapy (CTX, paclitaxel, and bevacizumab) for left-sided breast cancer. **a** Contrast-enhanced computed tomography (CT) reveals a smooth hepatic margin before CTX. **b** A nodular contour is observed in the lateral liver segment on contrast-enhanced CT performed 7 months after the initial CTX. It is difficult to differentiate pseudocirrhosis from liver cirrhosis on a single image alone

After chemotherapy-induced hepatic toxicity, patients with liver metastases from breast cancer are most likely to experience diffuse morphological changes [19, 20]. However, it may also occur in patients with other types of cancer. Pseudocirrhosis can occur after hormonal therapy and anti-HER2 therapy [19]. Two hypotheses exist regarding the underlying mechanisms: (i) Chemotherapy causes a reduction in the liver metastases size. The liver then develops severe desmoplastic fibrosis, resulting in diffuse morphological alterations. (ii) Chemotherapy causes nodular regenerative hyperplasia of the liver [4]. Small regenerative nodules are dispersed throughout the liver and can cause diffuse morphological changes. Pseudocirrhosis imaging characteristics include liver surface nodularity, multifocal capsular retraction (Fig. 8), decreased liver size, caudate lobe enlargement, and signs of portal hypertension, including portal-systemic collateral pathways, splenomegaly, or ascites [4, 20]. Although the imaging findings resemble cirrhosis, the pathological characteristics of cirrhosis are not evident. Pseudocirrhosis is frequently associated with a poor prognosis in affected patients [19, 20].

##### Chemotherapy-associated steatohepatitis

**Fig. 9:**
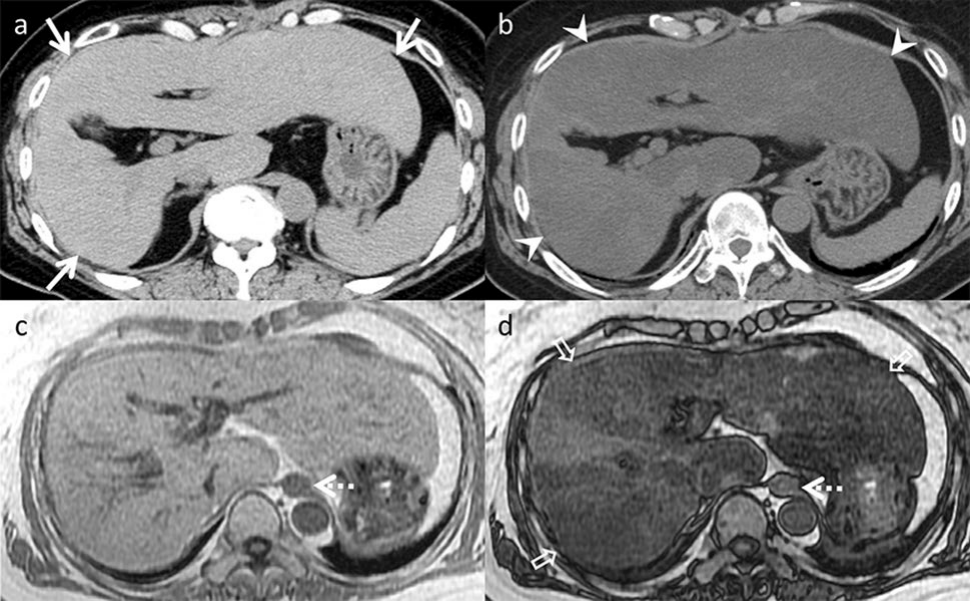
Chemotherapy-associated steatohepatitis in a 62-year-old woman with ascending colon cancer, who was undergoing chemotherapy (CTX), including irinotecan for left adrenal metastasis (dotted arrow). **a** Unenhanced computed tomography (CT) demonstrates normal liver attenuation (CT value, 55 HU) before CTX (arrows). **b** Decreased liver attenuation (21 HU) is observed on unenhanced CT performed seven months after CTX initiation (arrowheads). Additionally, compared to the **c** in-phase chemical shift imaging (CSI), a heterogeneous signal drop (open arrows) is seen on the **d** opposed-phase CSI. No abnormal liver function or hepatitis was indicated by hematological data or clinical symptoms. It is difficult to differentiate chemotherapy-associated steatohepatitis from simple liver steatosis on a single image alone

Chemotherapy-associated steatohepatitis ranges from simple hepatic steatosis to steatohepatitis and is mostly observed in patients receiving 5-fluorouracil and irinotecan treatment [11]. Patients receiving irinotecan-based regimens have a 3.45-fold greater risk of developing steatohepatitis than those who have never received chemotherapy [4]. The development of this condition is associated with a disturbance in lipid metabolism and lipoprotein synthesis alterations in hepatocytes, causing an increase in hepatocellular lipid content. Chemotherapy-associated steatohepatitis increases postoperative morbidity and mortality risks [4, 11]. The liver parenchyma demonstrates reduced attenuation when evaluated using a CT. Decreased tumor-to-liver contrast during the portal venous phase can impede metastatic detection. MRI has a higher detection sensitivity for moderate steatosis. Identifying and quantifying the fatty infiltration extent within the liver are facilitated by techniques including CSI (Fig. 9) and proton density fat fraction [4, 11]. Chemotherapy-associated steatohepatitis is generally reversible when the causative chemotherapy is discontinued [21]. While it has been associated with poor postoperative prognosis [11], drug interruption should be carefully considered, weighing risks and benefits.

##### Amiodarone deposition in the liver

**Fig. 10:**
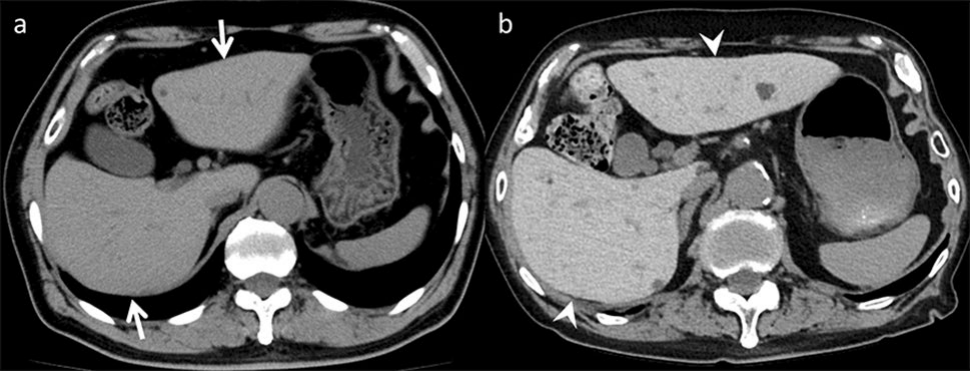
Amiodarone deposition in the liver of an 82-year-old man with ventricular tachycardia (VT), who was undergoing amiodarone therapy for VT. **a** Unenhanced computed tomography (CT) demonstrates normal liver attenuation (CT value, 57 HU) before amiodarone therapy (arrows). **b** Increased liver attenuation (97 HU) is observed on unenhanced CT performed nine years after amiodarone initiation (arrowheads). No abnormal liver function or hepatitis was indicated by hematological data or symptoms. It is difficult to differentiate amiodarone deposition from iron overload on a single image alone

Amiodarone, an effective antiarrhythmic medication, has been linked to liver-related adverse effects, including steatohepatitis and cirrhosis [22]. Increased liver attenuation on CT is a characteristic finding associated with long-term use of amiodarone [23] (Fig. 10). This increase in attenuation is caused by amiodarone accumulation in the liver as it contains two iodine atoms that account for approximately 40% of its molecular weight. Amiodarone is more likely to accumulate in the liver than in skeletal muscle or spleen. There is no correlation between the cumulative amiodarone dose and the change in liver density after administration. Previous reports have shown that CT values of the liver may not indicate the severity of liver damage [24]. Discontinuing amiodarone may lead to a decrease in liver attenuation on CT [25].

##### Secondary iron overload

**Fig. 11:**
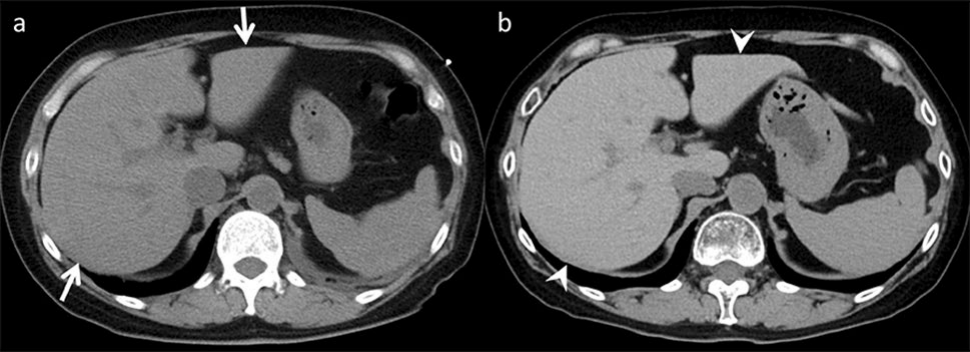
Secondary iron overload in a 61-year-old woman with malignant lymphoma, who was undergoing repeated blood transfusions for chemotherapy-induced myelosuppression. **a** Unenhanced computed tomography (CT) demonstrates normal liver attenuation (CT value, 46 HU) before the blood transfusions (arrows). **b** An increase in liver attenuation (75 HU) is observed on unenhanced CT performed 1.5 years after initiating blood transfusions (arrowheads). No hematological data or symptoms indicated abnormal liver function or hepatitis. It is difficult to differentiate iron overload from amiodarone deposition on a single image alone

Iron overload can result from excessive iron absorption due to repeated blood transfusions, excessive oral iron consumption, and the use of parenteral nutrition elements. Each unit of transfused blood contains 200–250 mg of iron, and a substantial risk arises when more than 10–20 blood units are transfused [26]. Parenteral nutrition preparations may include iron, manganese, zinc, copper, and iodine [27]. An increase in liver attenuation on CT scans may indicate hepatic iron deposition (Fig. 11). Excess iron is diagnosed based on elevated serum ferritin, iron, and transferrin saturation levels. MRI is the preferred noninvasive diagnostic modality for iron overload and is widely accepted. Various techniques for iron quantification using MRI exist, with R2*-based liver iron concentration considered the most feasible and supported by substantial evidence [28]. Iron chelation therapy, involving medications, such as deferasirox, deferoxamine, and deferiprone, removes excess iron from the body and treats iron accumulation [26, 29].

### Pancreas

#### Drug-induced pancreatitis

**Fig. 12:**
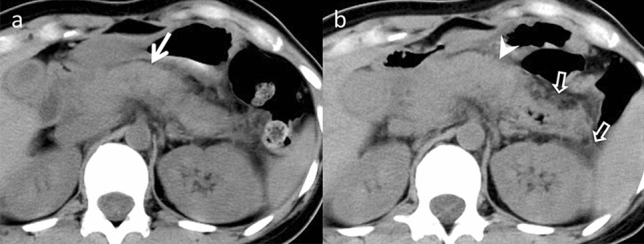
Drug-induced pancreatitis in a 30-year-old woman with abdominal pain, who was undergoing L-asparaginase therapy for acute lymphocytic leukemia. **a** Unenhanced computed tomography (CT) demonstrates the normal pancreas size before L-asparaginase therapy (arrow). **b** Pancreatic enlargement (arrowhead) and retroperitoneal fat stranding (open arrows) are observed on unenhanced CT performed one week after the L-asparaginase initiation. On imaging, acute pancreatitis from other causes is a differential; however, both are nonspecific and difficult to distinguish on imaging alone

Drug-induced pancreatitis caused by drugs administered for therapeutic purposes, presents as acute pancreatitis without progressing to chronic pancreatitis. Its frequency ranges from 0.1 to 2% of all cases of acute pancreatitis. While most cases have a good prognosis and are mild, there are severe and fatal cases [30]. Drug-induced pancreatitis occurs due to pancreatic duct obstruction, acinar cell damage, and dysfunctional intracellular transport, with drug-specific toxicity and patient allergic reactions as possible mechanisms [31]. Pancreatitis from drug-specific toxicity typically develops within 24 h, although it is extremely rare. Allergic reactions to drugs are considered the main pathogenesis of drug-induced pancreatitis, with onset occurring 1–6 weeks after administration, often within 30 days [32]. Various drugs have been reported as causative agents, including angiotensin-converting enzyme inhibitor, 5-aminosalicylic acid, and L-asparaginase [30]. Imaging findings resemble those of typical acute pancreatitis, including pancreatic parenchymal enlargement, indistinct pancreatic margins, and surrounding retroperitoneal fat stranding (Fig. 12). Treatment involves discontinuation of suspected drugs are the primary approach, followed by managing acute pancreatitis based on disease severity.

#### Pancreatic enlargement after epoprostenol therapy

**Fig. 13:**
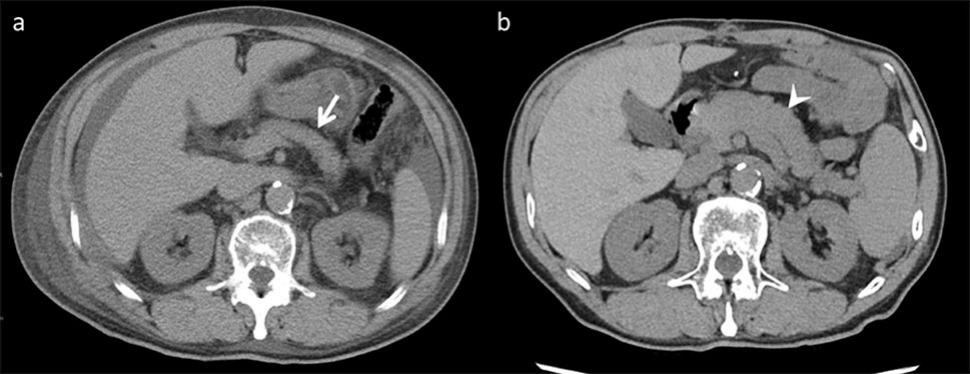
Pancreatic enlargement in an asymptomatic 70-year-old man with pulmonary arterial hypertension (PAH), who was receiving epoprostenol therapy for PAH. **a** Unenhanced computed tomography (CT) demonstrates the normal pancreas size before epoprostenol therapy (arrow). **b** Pancreatic enlargement is observed on unenhanced CT performed four months after the epoprostenol initiation (arrowhead). On imaging, acute pancreatitis is a differential; however, the absence of retroperitoneal fat stranding is a key to distinguishment

Epoprostenol, also known as prostaglandin I2 (PGI2), is a prostacyclin analog that induces vasodilation and inhibits platelet aggregation. It is a valuable therapeutic option for severe pulmonary arterial hypertension (PAH) [33]. While thyroid enlargement is a well-known adverse effect in some PAH patients treated by epoprostenol, reports of pancreatic enlargement following epoprostenol therapy are limited [34] (Fig. 13). Typically, the absence of elevated pancreatic enzymes and pancreatitis-related symptoms helps distinguish it from acute pancreatitis. Studies in a dog model have shown that PGI2 increases pancreatic blood flow, resulting in dose-dependent pancreatic vasodilation [35]. This increased blood flow may contribute to pancreatic enlargement.

### Biliary system

#### Ceftriaxone (CTRX)-associated gallbladder pseudolithiasis

**Fig. 14:**
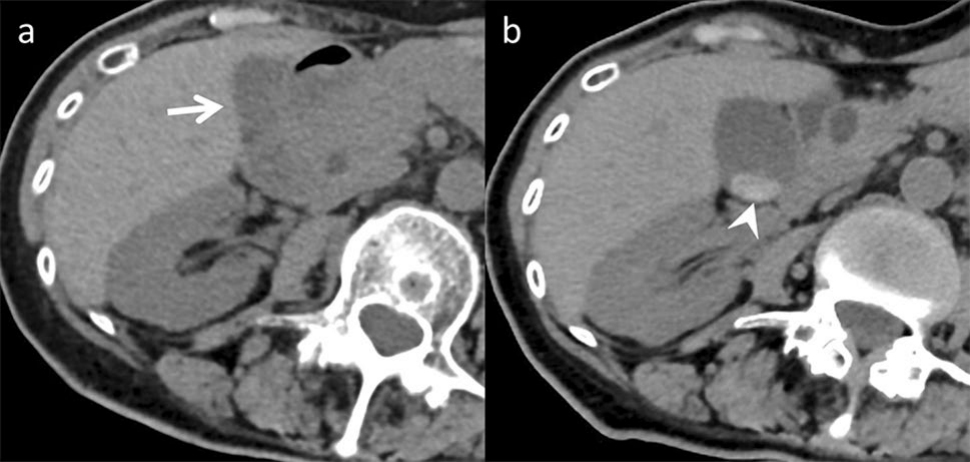
Ceftriaxone (CTRX)-associated gallbladder pseudolithiasis in an asymptomatic 65-year-old woman with diabetes mellitus, who was undergoing antibiotic therapy for iliopsoas abscesses, including CTRX. **a** Unenhanced computed tomography (CT) demonstrates no gallbladder abnormalities before CTRX initiation (arrow). **b** A high-attenuation object (stone pattern) is observed in the gallbladder on unenhanced CT performed two weeks after CTRXinitiation (arrowhead). No examination using contrast material between the CT examinations was performed. It is difficult to differentiate pseudolithiasis from gallbladder stones on a single image alone

CTRX, a widely used third-generation cephalosporin, is associated with an increased incidence of pseudolithiasis in children [36]. However, studies have also reported CTRX-associated biliary pseudolithiasis in adults. Risk factors for pseudolithiasis include limited activity or increased dehydration risk in elderly individuals, a cumulative CTRX dose exceeding 19 g, and prolonged CTRX treatment [37]. Pseudolithiasis formation occurs within 2–48 days after initiating CTRX treatment [38]. Although often asymptomatic, pseudolithiasis can lead to complications, such as cholecystitis, cholangitis, and pancreatitis [36]. Common CT findings include sludge, stones (Fig. 14), or a combination both. The CT value for pseudolithiasis ranges 58–202 HU, with a median of 83 HU. Additionally, CTRX may cause gallbladder enlargement and stone formation in the common bile duct. Discontinuing CTRX therapy typically results in resolution of pseudolithiasis within approximately 69 days [38].

### Multi-organ

#### Immune-related adverse events (irAEs)

**Fig. 15:**
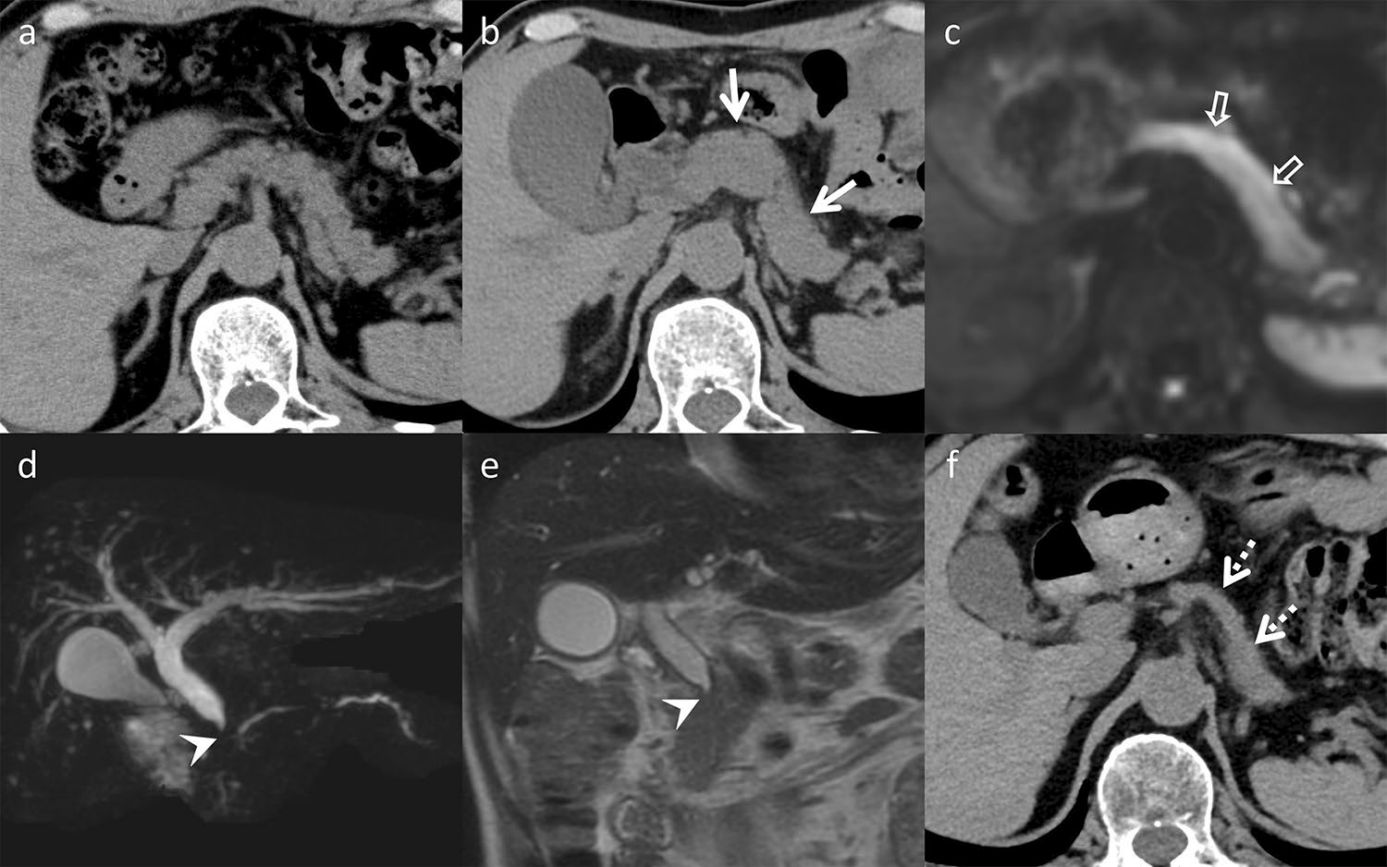
Immune-related adverse events in an asymptomatic 65-year-old man, who was undergoing chemotherapy (CTX, nivolumab) for bone metastases from renal cell carcinoma after a nephrectomy. **a** Unenhanced computed tomography (CT) reveals no pancreatic abnormalities one year following the initial CTX. Two years after the initial CTX, pancreatic enlargement, loss of lobulation (arrows) on **b** unenhanced CT, and diffuse hyper-intensity on **c** diffusion-weighted imaging (open arrows) are detected. Additionally, obstruction and wall thickening of the common bile duct are noted on **d** magnetic resonance cholangiopancreatography and **e** T2-weighted imaging (arrowheads). The pancreas is atrophic after steroid therapy on **f** unenhanced CT (dotted arrows). On imaging, immunoglobulin G4-related disease is a differential; however, it is difficult to differentiate immune-related adverse events from immunoglobulin G4-related disease on imaging alone

ICI adverse effects may result from impaired self-tolerance and affect every organ in the body. Common irAEs manifestations associated with ICI are gastrointestinal, endocrine, and dermatological toxicities [39]. However, ICIs can cause hepatic, pancreatic, and biliary toxicities. Hepatitis is a common hepatic irAE affecting 1–11% patients and often presents without discernible symptoms. CT and MRI typically reveal liver parenchymal heterogeneous enhancement, periportal edema, and portal lymphadenopathy [40, 41]. One to 13 percent cases exhibit pancreatitis. CT and MRI of patients with pancreatic irAEs reveal diffuse pancreatic enlargement and peripancreatic inflammation. Subsequent imaging reveals parenchymal atrophy and loss of normal lobulation following symptom resolution [39] (Fig. 15). Cholecystitis and cholangitis caused by ICIs are uncommon, affecting less than 3% patients. CT and MRI characteristics of biliary irAEs include gallbladder distension, gallbladder as well as bile duct wall thickening, bile duct narrowing or obstruction, and pericholecystic inflammation [40] (Fig. 10).

#### Methotrexate-associated lymphoproliferative disorders (MTX-LPD)

**Fig. 16:**
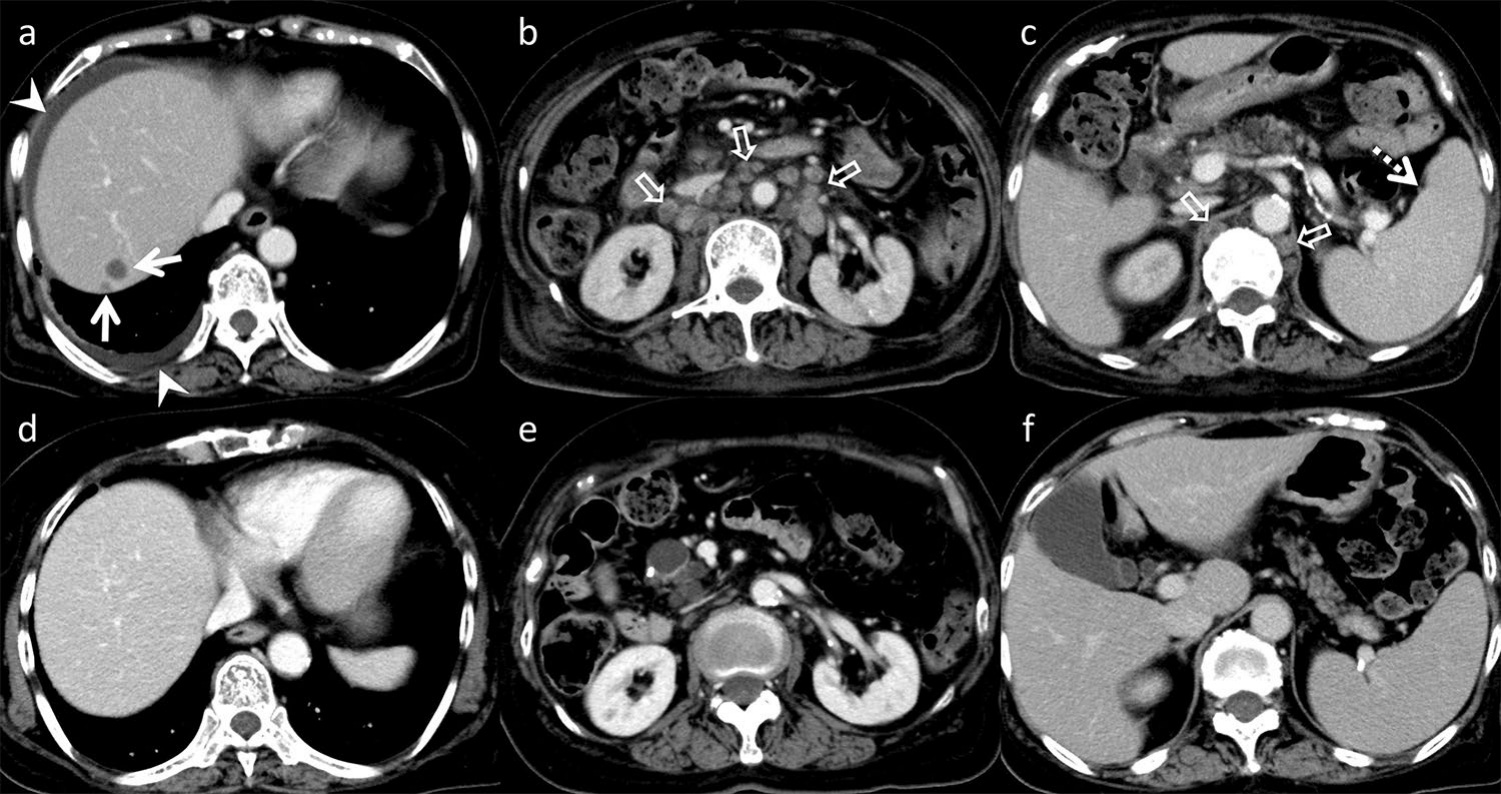
Methotrexate-associated lymphoproliferative disorder in a febrile 75-year-old man, who was taking methotrexate for rheumatoid arthritis. **a**–**c** Hypovascular hepatic nodules in S7 (arrows), ascites and right pleural effusion (arrowhead), lymphadenopathy (open arrows), and splenomegaly (dotted arrow) are observed on initial contrast-enhanced computed tomography (upper row). **d**–**f** These findings subsided four months after the withdrawal of methotrexate (lower row). It is difficult to differentiate methotrexate-associated lymphoproliferative disorder from malignant lymphoma on a single image alone

LPD are characterized by uncontrolled lymphocyte production, resulting in monoclonal lymphocytosis, lymphadenopathy, and bone marrow infiltration [42]. This disorder is often associated with MTX administration, suppressing immune surveillance [43]. Epstein–Barr virus is regarded as the primary causative agent in most MTX-LPD cases [42]. MTX-LPD is found in lymph nodes and other organs, with frequent extra-nodal involvement in the lungs, skin, and soft tissues, but uncommon involvement in the liver. Pancreatic lesions are rarely reported. However, CT and MRI imaging reveal hypo-vascular liver masses and multiple lymphadenopathies [43] (Fig. 16). The primary treatment for MTX-LPD is the discontinuation of MTX administration. Chemotherapy may be required to effectively manage the disease if the condition does not improve [42, 43].

## Conclusion

Diagnosing drug-induced hepatobiliary and pancreatic disorders through imaging can be challenging, but some conditions exhibit characteristic findings on CT or MRI. For instance, SOS is identifiable by the hypo-intense reticular hepatic parenchyma during hepato-biliary phase. While many other conditions have nonspecific imaging findings, their recognition can aid in confirming the patient's treatment history and preventing mismanagement.
